# Monoclonal antibody or aspirin desensitization in NSAID-exacerbated respiratory disease (N-ERD)?

**DOI:** 10.3389/falgy.2023.1080951

**Published:** 2023-04-12

**Authors:** Dorien Van Broeck, Brecht Steelant, Glenis Scadding, Peter W. Hellings

**Affiliations:** ^1^ Department of Microbiology, Immunology and Transplantation, KULeuven, Leuven, Belgium; ^2^ Royal National ENT Hospital and Division of Infection and Immunity, University College, London, United Kingdom; ^3^ Department of Otorhinolaryngology, Head and Neck Surgery, University Hospitals Leuven, Leuven, Belgium

**Keywords:** NSAID-exacerbated respiratory disease, aspirin desensitization, aspirin, dupilumab, benralizumab, omalizumab, mepolizumab, reslizumab

## Abstract

Nonsteroidal anti-inflammatory drug (NSAID)-exacerbated respiratory disease (N-ERD) is a clinical syndrome characterized by nasal polyposis, asthma, and intolerance to aspirin/NSAID. It affects approximately 15% cases of severe asthma, 10% of nasal polyps and 9% of rhinosinusitis. N-ERD results in associated asthma exacerbations, oral corticosteroids bursts, corticosteroid-dependent disease, and multiple endoscopic sinus surgeries. Unknown influences cause polyp epithelium to release alarmins, such as IL-33 and TSLP. These cytokines activate lymphoid cells, both Th2 and ILC2, to release cytokines such as IL5, IL4 and IL13, resulting in complex type 2 inflammation involving mast cells, eosinophils and platelets. Arachidonic acid released from such cells is metabolized into mediators. N-ERD is characterized by an imbalance in eicosanoid levels, especially CysLTs, PDG and PGE2. Patients with N-ERD present nasal symptoms (congestion, hyposmia/anosmia, nasal discharge) and lower airways symptoms (cough, sneezing, shortness of breath, chest tightness), anosmia, severe hyposmia as well as severe asthma which impacts the quality of life in this disease and leads to safety concerns in patients daily lives. Despite the variety of treatment strategies, the likelihood of recurrence of symptoms is high in patients with N-ERD. The most important strategies for treating N-ERD are listed as following: drug therapies, aspirin desensitization, monoclonal antibodies and other therapies associated. N-ERD treatment remains a major challenge in the current situation. Selecting the appropriate patient for aspirin desensitization, monoclonal antibodies or both is essential. This review provides an overview on aspirin desensitization and biologics in N-ERD and might help in decision making from both the perspective of the physician and patient. Patient characteristics, safety, efficacy, health care costs, but also patient preferences are all factors to take into account when it comes to a choice between biologics or aspirin desensitization.

## Introduction

NSAID-exacerbated respiratory disease (N-ERD) is an inflammatory and complex disease, characterized by the clinical triad of asthma, chronic rhinosinusitis with nasal polyps, and the development of respiratory reactions to nonsteroidal anti-inflammatory drugs (NSAIDs). Treatment of N-ERD combines therefore the standard care for the relief of asthma and chronic rhinosinusitis symptoms (1). Up to 16% of patients with CRSwNP have intolerance to NSAID, while 7%–15% of asthma patients are affected (2). For the upper airways, the established guidelines recommend first nasal douches, topical glucocorticoids (mometasone, budesonide, fluticasone) and if not sufficient, antihistamines in allergic individuals, leukotriene modifiers and systemic glucocorticoids are added in a stepwise fashion. Based on the overproduction of cysteinyl-leukotrienes in N-ERD, it is worthwhile to consider zileuton, a 5-lipo-oxygenase inhibitor, which has also a significantly effect on LTE4 as it partially blocks the formation of all cysteinyl leukotrienes, in contrast to the CysLT1 receptor antagonists montelukast, zafirlukast, and pranlukast (3). The additional advantage of these agents is the protection from severe respiratory reactions after accidental ingestion of NSAID (1). Leukotriene-modifying agents are used in one of the last steps of the treatment guidelines for the lower airways, when corticosteroids in combination with long-acting beta-agonists fail to bring relief. In patients with N-ERD, asthma remains often uncontrolled and nasal polyps and sinusitis often return, even after repeated courses of systemic corticosteroids and multiple sinus surgeries (4). In this phase, the physician is left with two treatment options to attack the persistent airway inflammation: aspirin desensitization or biological therapy. However, no direct trials with aspirin desensitization and biologic therapy in N-ERD patients exist which makes it difficult to choose. This review provides an overview on aspirin desensitization and biologics in N-ERD and might help in decision making from both the perspective of the physician and patient. Patient characteristics, safety, efficacy, health care costs, but also patient preferences are all factors to take into account when it comes to a choice between biologics or aspirin desensitization.

Aspirin desensitization has been introduced after the following two observations were reported: firstly, there is a time phase after aspirin ingestion wherein repeat intake does not lead to adverse reactions. This time phase is known as the refractory period and can range from 24 h to several days (5). Secondly, the clinical condition of patients with aspirin intolerance improves during this refractory period: Stevenson et al. who investigated the refractory period of two patients with aspirin intolerance, observed an increase in FEV1, a reduction in oral corticosteroid intake and a healthy lining of the nasal membrane during nasal examination after six months of daily aspirin use. Furthermore, the daily intake of aspirin resolved the nasal symptoms of these two patients (6). To further investigate this phenomenon, several open and controlled trials were conducted, first on a short-term basis followed by long-term studies. In this way aspirin challenge followed by daily aspirin intake was introduced as a possible treatment option for N-ERD. Since then, several desensitization protocols have been developed, both by systemic and topical route (7).

Although the mechanism underlying the benefit of aspirin desensitization in patients with aspirin intolerance is still not fully understood, there seems to be a particular role for “Prostaglandin D2” (PGD2) and “Cysteinyl leukotrienes” (CysLTs) (2, 8). PGD2 first increases during desensitization, whereas its’ concentration decreases during the daily intake of aspirin (2). If the initial increase of PGD2 is too high, patients might be unable to tolerate aspirin desensitization. If not, the subsequent decrease of PGD2 during daily aspirin intake results in reduced expression of CysLT receptors, inflammatory cell infiltrate and concentration of type 2 cytokines like IL-4, IL-5 and IL-13, which are released by type 2 innate lymphoid cells (2, 8, 9). As type 2 inflammation (which is associated with higher disease severity and recurrence) seems to be the predominant endotype in 85% of CRSwNP in the Western world, biologics targeting IL-4, IL-5 and IL-13 in severe asthma became novel treatment options for CRSwNP, especially in the case of co-morbid asthma and N-ERD (10, 11).

## From the perspective of the physician

### What characterizes patients for a treatment with either monoclonal antibodies or aspirin desensitization?

Currently, no biomarkers are known to predict the response of the patient to aspirin desensitization or biological therapy, but patient's characteristics can already point into a direction (1). For example, when co-morbidities like cardiovascular disease or inflammatory disorder are present, the medical need for tolerance to aspirin/cyclo-oxygenase 1 (COX-1) therapy is obvious and favours aspirin desensitization. This enables the N-ERD patient to take prophylactic daily aspirin as antiplatelet therapy or to take daily NSAIDs as painkillers to relief the pain associated with a rheumatologic condition (7).

On the other hand the patient's history can make aspirin desensitisation not the preferred option due to the presence of some absolute or relative contra-indications to aspirin, like a history of peptic ulcers, eosinophilic esophagitis (which can also be associated with N-ERD), renal impairment, taking anticoagulant/antiplatelet therapy and history of bleeding disorders (1, 12, 13). These patients are at elevated risk for aspirin-related adverse effects as long-term aspirin desensitization diminish synthesis of gastric prostaglandin (PGI2) formation and causes inadequate repopulation of gastric mucosal cells, leading to gastric pain or ulcer, and as aspirin inhibits platelet function by acetylating platelet cyclo-oxygenase (3). According to White et al. 2020 between 10% and 15% of the N-ERD patients will be unable to remain on aspirin therapy due to the gastrointestinal side effects or bleeding/bruising (1). Remarkably, black and Latino patients are more prone than white patients to fail to tolerate the initial aspirin desensitization due to persistent gastrointestinal symptoms (2).

The necessity to evaluate the risks and comorbidities in the choice for oral aspirin desensitisation, becomes more important when the N-ERD patient becomes older. Recent clinical research suggests that daily oral aspirin is associated with a very small but significantly higher risk of major bleeding and haemorrhage and possibly a higher all-cause mortality in older adults, suggesting biological therapy as alternative treatment, although data for biologics in advanced age are lacking (2, 14). The treating physician should therefore be continuously attentive for aspirin-related adverse effects and asses the benefit/risk ratio of aspirin desensitization regularly as patients' history can change.

In line with this, some contra-indications for oral aspirin desensitization are temporary, like for example pregnancy and planned sinus surgery. In pregnant N-ERD patients, the intake of aspirin doses larger than 81 mg daily can contribute to premature closure of the ductus arteriosus and increases the risk for maternal and foetal bleeding. Therefore, oral aspirin desensitization should not be initiated or discontinued during pregnancy (2, 13). There is some consensus amongst the Rhinology community that aspirin desensitization should be started after sinus surgery, because of the potential for increased intraoperative bleeding and associated decrease in intraoperative visualization after aspirin intake, but also because of better clinical outcomes of aspirin desensitization after debulking the inflammatory nasal polyp tissue (2). Until now, it is unclear whether initiating biologics immediately after surgery would have a better clinical effect, but in every case, however, surgery is not a contra-indication.

Another important factor to consider is the extent to which the patient can adhere to the treatment. Patients on aspirin treatment after aspirin desensitisation (ATAD) missing their dose for more than two days will gradually regain their intolerance and will need further desensitization (2, 15).

Finally, there are some prerequisites associated with the start of aspirin desensitization or biological therapy. For aspirin desensitization, it is recommended that the patient has stable/controlled asthma (FEV1 > 70% predicted) before initiating the therapy to prevent further exacerbation of patients’ asthma (13, 15). For biologics, governments define criteria that need to be met for reimbursement of biologics. Clinical criteria to start biological therapy include, but are not limited to, evidence of type 2 inflammation, need for systemic corticosteroids, impaired quality of life, previous sinus surgery, failure of treatment with nasal corticosteroids, significant loss of smell and co-morbid asthma (16, 17). In N-ERD patients, asthma is not always present from the start according to a distinctive pattern characterized by a sequence of symptoms with first persistent rhinosinusitis. Asthma can thus be non-existent at the time of diagnosis of N-ERD (18). Interestingly, research has shown that even patients without asthma can benefit from aspirin desensitization (19).

### Which effect is to be expected?

The biologics omalizumab, mepolizumab and dupilumab are approved for use in patients with severe, uncontrolled chronic rhinosinusitis as add-on therapy to nasal corticosteroids. Their effect sizes seem large enough to reflect a major reduction in symptom burden as experienced by patients suffering from refractory CRSwNP (11). Benralizumab is not yet approved, but the OSTRO Phase III trial which met both co-primary endpoints with a safety profile consistent with the known profile of the medicine, is completed (20). An important side note is that data for N-ERD patients are generated by post-hoc analysis as they constituted only a subgroup of the whole study population. The percentage of participants with N-ERD within the group of patients with CRSwNP was 27% for mepolizumab, 28% of the overall population for dupilumab, 30% in each treatment arm for benralizumab and between 17% and 39% for the two trials with omalizumab (15). A second site note is that diagnosis of N-ERD based on history alone is not always reliable (21), and that provocation tests are necessary to have a confirmed diagnosis of N-ERD. Extrapolated data show that patients with N-ERD responded equally well compared to the aspirin-tolerant subgroup on the treatment with reduction of polyp size. In addition, N-ERD patients responded equally well on the dupilumab treatment with improvement in sense of smell and equally well in nasal blockage score on the benralizumab treatment. Dupilumab is the only biological showing a difference between aspirin-tolerant and aspirin-intolerant CRSwNP patients. More specifically, N-ERD patients reported significantly greater improvement in nasal congestion and SNOT-22. The efficacy of mepolizumab, benralizumab and dupilumab in patients with N-ERD for the treatment of asthma is also to be expected at least equally and even potentially superior given the fact that outcomes improved with higher numbers of peripheral eosinophils (15).

Since the first introduction of aspirin desensitization by Widal and associates in 1992 (3), only 5 double-blind placebo-controlled trials were conducted to assess the efficacy and safety of ATAD, totalling only 163 patients. The low number of double-blinded placebo-controlled trials can be explained by the fact that blinding of desensitization is difficult since patients in the active arm will experience a worsening of their symptoms during desensitization while those patients in the placebo arm will not. Alternatively, true desensitization with aspirin can be performed in all patients before randomization in the active or placebo arm (2, 15). Furthermore, there is no interest of the pharmaceutical industry in the repurposing of such an old molecule as aspirin for a new indication besides pain, fever, and prevention of blood clots. In cross-over trials from the early 1980s, 67% of the 25 patients noted improvement in nasal symptoms, while only half of the patients experienced improvement in their asthma symptoms. In general, most patients reported not only improvement in upper but also in lower respiratory symptoms, as well as overall disease control. Taken together, including one meta-analysis focusing on ATAD, the American Academy of Asthma Allergy & Immunology considers aspirin treatment after aspirin desensitization a unique treatment option for patients with N-ERD. Interestingly, a new systemic review and meta-analysis compared the efficacy and safety of ATAD with 8 other mAbs. More specifically, besides the 5 RCTs about ATAD, they included 24 RCTs evaluating 7 different mAbs. Comparisons among biologics and aspirin desensitization show with moderate to high certainty that dupilumab is among the most beneficial for 7 of 7 patient-important and surrogate outcomes, omalizumab for 2 of 7 (HRQoL and sinusitis symptoms), mepolizumab for 1 of 7 (sinusitis symptoms), and aspirin desensitization for 1 of 7 (sinusitis symptoms) (22). Aggregation of the data about aspirin desensitization is difficult because of the differences in study protocol (total daily aspirin dose, length of treatment) and primary endpoints (14). Moreover, most of the studies performed have a retrospective study design. The longest retrospective study has shown that 85% of patients were still taking daily aspirin 10 years after desensitization because they felt that the aspirin was “very” or “extremely” helpful in controlling their sinonasal and asthma symptoms as well as in improving their quality of life (2, 13). Although study results about the effect of ATAD on the need for revision surgery are conflicting, there is a consensus that ATAD is beneficial in the prevention of regrowth of nasal polyps after debulking surgery, rather than in the reduction of existing polyp burden (15). Finally, not unimportantly, ATAD improves N-ERD patients’ intolerance to alcohol (13).

### How safe are biologics and ATAD?

Long term safety data for the biologics are lacking, especially in the field of allergy and rhinology. In the short term, the use of biologics seems to be relatively safe, with a drop-out rate of less than 5% for most of the published phase 3 studies (15). According to the meta-analysis of Oykhman et al. comparing 24 RCTs, the occurrence of adverse events with dupilumab, omalizumab, mepolizumab, benralizumab and reslizumab differed little from placebo (22).

An issue associated with dupilumab treatment might be the risk of increased blood levels of eosinophils in roughly 10% of patients. At circa 16 to 20 weeks after starting the treatment, eosinophil levels were 10% higher compared with baseline. Several patients (<5%) needed a rescue treatment to continue on dupilumab for their asthma or chronic rhinosinusitis (15). Permanent discontinuation of dupilumab was needed in 7 adverse events of eosinophilia compared to 1 in the placebo arm (23). However, peripheral eosinophilia is also associated with high dose aspirin therapy in N-ERD (2).

Although ATAD was central to the management of N-ERD for the past 3 decades, safety data on high dose aspirin are limited due to the small size trials and the short study duration (14). One retrospective study was formally analysed to determine the rate of major complications associated with aspirin desensitization and maintenance therapy that resulted in discontinuation (24). Of the 109 N-ERD patients, included from July 2016 to February 2019, who underwent ESS with subsequent ATAD, 18 patients discontinued the therapy due to gastritis (9, 8.2%), GI bleeding (1, 0.92%), anaphylaxis (1, 0.92%), exacerbation of upper airway symptoms (1, 0.92%), recurrent epistaxis (1, 0.92%), exacerbation of lower airway symptoms (4, 3.7%) and cutaneous reaction (nummular rash) (1, 0.92%). These results are in line with the report of the working group of the American Academy of Allergy, Asthma & Immunology, stating that gastritis is the most common reason for discontinuation of maintenance therapy. Although the bleeding risk while taking high doses of aspirin has not specifically been studied in N-ERD, data extrapolated from its cardiovascular use shown a 50% increased risk of (gastro-intestinal) bleeding (2, 14). It is therefore of utmost importance to continuously monitor patients for aspirin-related side effects.

One alternative safer possibility is the use of topical intranasal lysine aspirin (LAS, the only soluble form) for both nasal challenge to diagnose N-ERD and subsequently as therapy, with gradual dose increase performed at home by the patient under close virtual supervision. Although a small blinded crossover trial with 16 mg did not improve symptoms (25), it did show a reduction in leukotriene receptors in the nasal mucosa. Open studies do suggest efficacy (26–29). The latest audit found higher nasal airflow and smell scores at each follow-up in N-ERD patients taking LAS (*p* < 0.001 and *p* = 0.048 respectively) compared tho those who were positive on challenge but did not continue therapy (30). No influence of LAS on pulmonary function measurements was observed in this study. Patient on intranasal LAS showed a lower rate of revision sinus surgery when compared to those who discontinued the treatment (*p* < 0.001). In addition, the doses now used are lower, at under 100 mg, than with oral ATAD, cause fewer gastric problems (3.8%) (31), and are more compatible with cardiac care since oral doses of over 100 mg (as necessary for oral ATAD) are associated with poor cardiovascular outcomes (32).

### What are the health care costs?

From the perspective of the health care system, less costly treatment options should be considered and tested before switching to complex and more expensive biological therapies. Aspirin is inexpensive, costing about $0,05 per tablet. The price for daily therapy is thus less than $100 per year. For the one-time cost of a desensitisation procedure, the price ranges from $1.700 to $3.000 (3, 33). In contrast, the cost of Th2 biological therapy is estimated to be $30.000 to $40.000 per patient per year. Given the fact that the age of onset of N-ERD is around 30 years and N-ERD is not remitting spontaneously, biological therapy cost multimillion dollars per patient per year (3, 13, 33).

Furthermore, there are currently no biosimilars for dupilumab, mepolizumab and omalizumab on the market to stimulate market competitiveness and to reduce the costs for the health care system. In Belgium, depending on the turnover threshold of the original biological, the price of a biosimilar can be up to maximum 47,18% lower than the original price of the original biological. Focusing only on the cost is, however, too short-sighted. The costs must be weighed against effectiveness, expressed by “quality-adjusted life year” (QALY), which stands for one year in perfect health.

In 2008, Shaker et al. investigated the cost-effectiveness of aspirin desensitization with subsequent aspirin therapy in moderate-to-severe N-ERD (34). According to their findings ambulatory desensitization for N-ERD could save $6.768 per QALY and across a wide range of assumptions, aspirin desensitization remained cost-effective with less than $50,000 per QALY saved. In their modelling of costs of asthma therapy, they did not include the potential of aspirin desensitization to reduce the need for omalizumab. Therefore, the abovementioned amounts are probably underestimating the cost saving effect of aspirin. Lysine aspirin is more expensive than aspirin tablets, but the process of challenge and initial desensitization is faster, taking half a day rather than 2–3 days of inpatient care, so the end result is cost-similar, very significantly lower than that of any biologic. A full-scale double- blind trial is needed.

Regarding the biologics, not surprisingly, cost-effective analyses are lacking, given their only recent approval for chronic rhinosinusitis. Anderson et al. reviewed in 2019 the current literature for asthma and the cost-efficacy of omalizumab, mepolizumab, reslizumab, benralizumab and dupilumab. The authors concluded that the prices should be reduced by a minimum of approximately 60% to achieve the cost-effectiveness threshold prices (35). In conclusion, there is something to be said for both biologics and aspirin desensitization. However, as the costs of aspirin desensitization are nothing compared to biologics, aspirin desensitization should be tried first when no clear contra-indication exists.

## From the perspective of the patient

To give patient-centred care, it is essential to involve patients in the choice between aspirin desensitization and biologics and to respect their preferences, needs and values. Some patients never want to experience that NSAID-induced reaction again and are terrified to start with aspirin desensitization. In a survey of clinics that threat patients with N-ERD, approximately half of the 109 patients were reluctant to go on aspirin therapy. When asked for the reason, 46 (45%) were concerned about taking aspirin long term, 28 (27%) were concerned about the safety, 19 (19%) said their physician did not recommend it, and 9 (9%) claimed the process was too expensive (24). On the other hand, patients might fear the effect of the biological on their immune system and the fact that only short-term safety data are known.

The Brigham and Women's Hospital surveyed 98 patients with N-ERD recruited from their registry to characterize patterns of medication use and efficacy (12). With 60% patients following ATAD and 49% on a respiratory biological, their study reflects well the current million-dollar question “monoclonal antibody or aspirin desensitization?”. While for ATAD the most common reason for discontinuation was “adverse effects”, “lack of efficacy” was the most common reason for stopping biological treatments. Interestingly, the reported efficacy of dupilumab was the highest, with 19 of the 26 patients ultimately transitioned to dupilumab and no patient switched from dupilumab to another biologic. These findings are in line with the results of a recent retrospective study concluding that, dupilumab leads to significantly higher rates of clinical improvement compared to anti-IL-5/IL-5R alpha and anti-IgE biologic therapies (36). Interestingly, the study of Bavaro et al. reported a difference in baseline serum IgE level of patients who responded better on treatment with dupilumab compared to mepolizumab. This observation might point towards the presence of different N-ERD-subendotypes (37), rendering the question which N-ERD-endotypes would benefit more from ATAD than biological therapy and vice versa. Using nasal mucus, Scott et al. identified 3 subendotypes of N-ERD characterized by (1) low inflammatory burden, (2) high type 2 cytokines, and (3) comparatively low type 2 cytokines and high levels of type 1 and 3 cytokines (38). Further research on biomarker-based endotyping and responder analyses are needed to guide the physician in choosing ATAD or biological as the best treatment option for the individual patient. Unfortunately, there are no head-to-head clinical trials comparing the efficacy of dupilumab with other respiratory biologics. In addition, because of the difference in the enrolment criteria, even the outcomes of the individual phase 3 biological trials are difficult to compare (39). Furthermore, there are no N-ERD-specific trials and although 30% of the dupilumab study population had N-ERD, N-ERD was not confirmed by provocation but history-based (15, 33). Only for dupilumab post-hoc analysis revealed an equally improvement in the sense of smell between aspirin-tolerant and -intolerant CRSwNP patients, making it a better choice for N-ERD-patients whose smell is impaired. According to the survey of Brigham and Women's Hospital, 40%–60% of the N-ERD patients reported improvement in the sense of smell during ATAD (12).

## Do we have to choose, can the patient receive both treatments?

**Figure 1:**
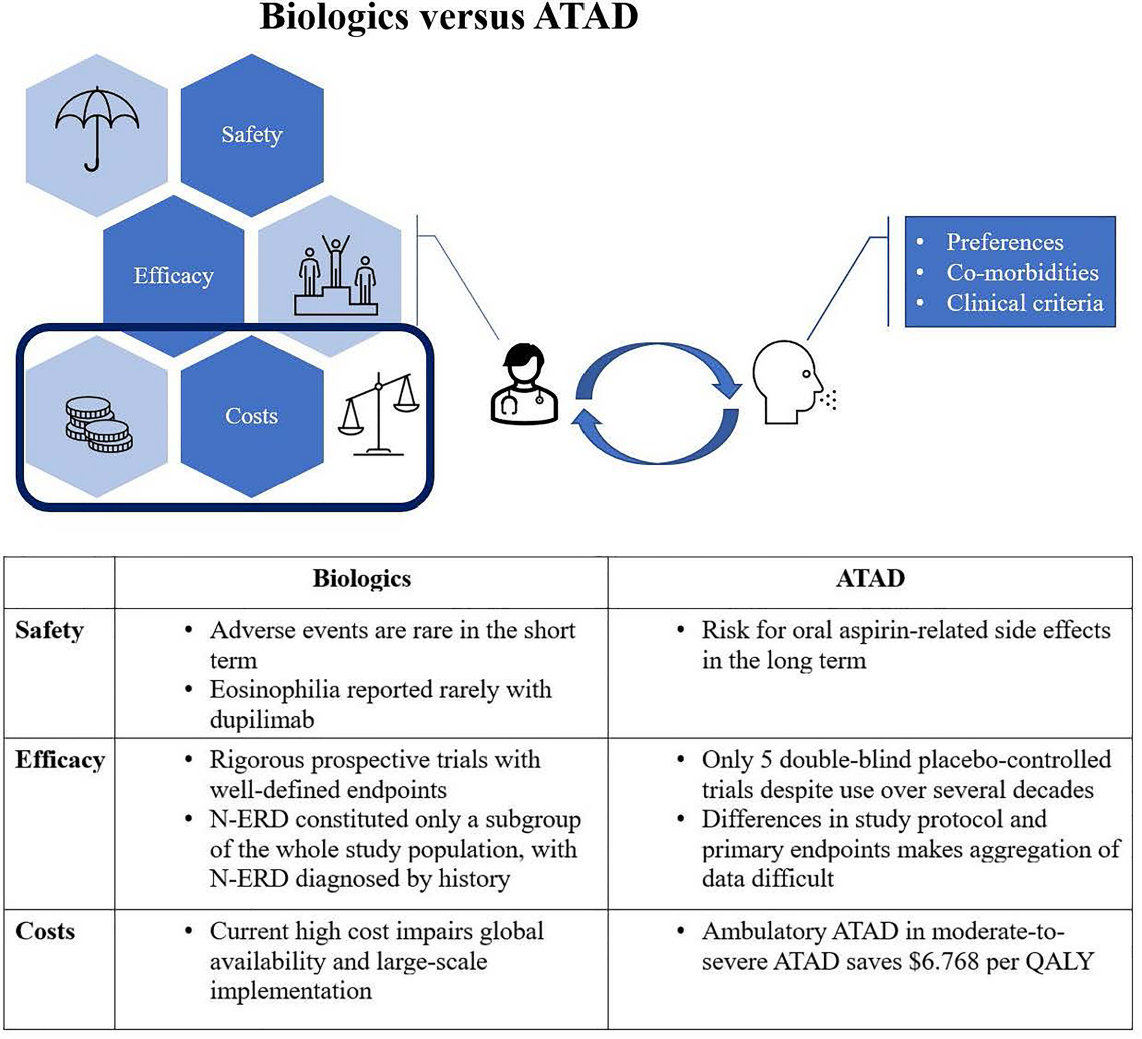
Different factors have to be weighed up continuously. Concerning health care costs, ATAD is clearly favourable. If the patient has the right profile, a trial of ATAD should therefore precede treatment with a biologic.

To help the physician in the choice between ATAD and biologics, White et al. reviewed three common situations in which timing plays a crucial role (1): (1) A newly diagnosed patient should undergo surgical debulking before starting step-up therapy to ATAD or biologics. (2) The patient with early polyp recurrence is an ideal candidate for ATAD as aspirin therapy might have the best effect in patients with recent surgical debulking. (3) In the case of chronic surgical failure, the risks and benefits of ATAD or a biological should be discussed, with guidance from patient preference, existing co-morbidities like cardiovascular diseases or need for treatment with NSAIDs, upcoming sinus surgery, etc. Similarly, Bucheit et al. proposed a treatment algorithm, offering biologics only following complete ESS and a trial of aspirin desensitization as biologics are associated with high costs and lack clear long-term safety data (40) (Figure 1).

Although comprehensive sinus surgery and ATAD provide excellent results in most patients, only a minority of patients will have an aspirin or surgical contraindication, are reluctant to surgery or aspirin desensitization, or do not have an excellent response to these coordinated therapies. In the study of the Brigham and Women's Hospital 24 of the 98 patients reported concurrent use of ATAD and a biologic (12). Indeed, the choice does not have to be dichotomous. In fact, ATAD and T2 biologics might have a synergetic effect while used in combination. T2 biologics help in difficult cases, when the response to ATAD is moderate, sufficiently efficacious to not warrant discontinuation, but not enough to preclude the need for further therapy. Furthermore, as immunotherapy can get worse before it gets better, T2 biologics might prevent exacerbation of the clinical manifestations of N-ERD during aspirin desensitization. Especially those N-ERD-subjects who have a distinct pruritic urticarial-like rash due to a manifest overproduction of prostaglandin D2 (PGD2) and leukotriene E4 (LTE4), might need the addition of a T2 biologic to tolerate aspirin desensitization or ATAD (33). In a randomized, double-blind, placebo-controlled study, a 16-week treatment with omalizumab was associated with a significantly greater likelihood for atopic N-ERD-subjects to have no respiratory reaction during desensitization (41). Contradictory, there were three cases of N-ERD patients who were on anti-IL-5-treatment with mepolizumab and still developed aspirin-induced reactions, including severe and systemic symptoms (42). However, some data are suggesting that the action of T2 biologics goes further than preventing exacerbated reactions, having the potential to induce aspirin-tolerance: out of 33 patients who underwent an aspirin challenge test before and after 6 months of anti-IgE therapy with omalizumab, 56% developed complete aspirin-tolerance (43). According to ClinicalTrials.gov, the Medical University of Vienna is currently running a study to test the efficacy of a 6-month treatment with dupilumab in inducing tolerance to aspirin evaluated by oral drug provocation testing (ClinicalTrials.gov Identifier: NCT04442256). The estimated study completion date is foreseen by the end of 2022.

## Conclusion

In summary, the advent of biologics has disrupted the established place of ATAD in the treatment of N-ERD and has left the physician with the one-million-dollar question of “how to position these two treatment options against each other? (Table 1)”. As patients with N-ERD constituted only a subgroup of the population with CRSwNP in phase III clinical trials, more specific trials about the efficacy of biologics in N-ERD are needed, as well as data about their cost-effectiveness. Real-world clinical studies on biologics in CRSwNP are now emerging and capturing endotypes, phenotypes and relevant biomarkers to answer questions such as which biologic gives the best result, which biomarker predicts responsiveness to therapy, and how long treatment should continue (44). The latter question is important to identify a disease-modifying effect of biologics in CRSwNP and in N-ERD. Several studies are already suggesting that not only can biologics be used as rescue treatment during aspirin desensitization, they might also introduce aspirin tolerance (41, 43, 45). However, more insight is needed into how aspirin desensitization provides benefit and why some patients benefit more from aspirin desensitization than others. Meanwhile, the physician has to rely on patient characteristics, patient preference and up-to-date data about efficacy and safety as elucidated in the sections above (Figure 1).

**Table 1:** Overview of the indications and contra-indications of biologics, ATAD and combined use (2, 7, 12, 15, 16).

	Biologics	ATAD	Combined use
Major considerations	Failed or contraindicated or unavailable trial of aspirin desensitization	Persistent sinonasal and asthma symptoms in a patient with N-ERD despite conventional medical and surgical therapy, especially when there is a need for aspirin-antiplatelet therapy or NSAIDs to treat chronic inflammatory conditions	Medium responders to ATAD; ATAD is sufficiently efficacious to not warrant discontinuation, but not enough to preclude the need for further therapy in the form of biologics
Additional considerations	Presence of bilateral nasal polyps and three of the following criteria if prior surgery: • Evidence of type 2 inflammation • Need for systemic corticosteroids (2 or more courses in the past year) • Significantly impaired quality of life • Significant loss of smell • Diagnosis of comorbid asthma If no history of surgery: 4 of the above criteria are required	Recent sinus surgery	To maintain cross-tolerance to other NSAIDs
			Severe asthma
Contra-indications	General contraindications for biological treatments, such as immunodeficiencies	General contraindications for aspirin, such as renal impairment	See the contra-indications for biologics or ATAD
	Patient-related factors such as noncompliance to therapy	Patient-related factors such as noncompliance to therapy	
	CRSsNP and lack of signs of type 2 inflammation	Poorly controlled asthma (in case of oral desensitization FEV1 needs to be ≥70 % of the patient's best and ≥1.5 L)	
	Cystic fibrosis	Significant nasal polyp burden at time of desensitization	
	Unilateral nasal polyps	Pregnancy	
	Mucoceles	History of eosinophilic esophagitis	
		History of gastric and/or peptic ulcer disease	
		History of a bleeding disorder or coagulopathy - additional risks for bleeding: • Prednisone use • Hypertension • Diabetes • Smoking • Male sex • Lower weight/body mass index • Use of other anticoagulants	
		History of anaphylaxis to NSAID	
